# Epistemic citizenship under structural siege: a meta-analysis drawing on 544 voices of service user experiences in Nordic mental health services

**DOI:** 10.3389/fpsyt.2023.1156835

**Published:** 2023-06-02

**Authors:** Faten Nouf, Jens Ineland

**Affiliations:** ^1^Department of Social Work, Umeå University, Umeå, Sweden; ^2^Department of Social Work, Faculty of Social Sciences, Umeå University, Umeå, Sweden

**Keywords:** mental health organizations, epistemic injustice, meta-analysis, Nordic countries, service user involvement, service user experiences, active citizenship

## Abstract

This paper presents a meta-analysis, drawing exclusively on qualitative research (*n* = 38), which contributes to findings on mental health service user experiences of received provisions and/or encounters in contemporary social and mental health services in the Nordic countries. The main objective is to identify facilitators of, and barriers to, various notions of service user involvement. Our findings provide empirical evidence regarding service users’ experiences of participation in their encounters with mental health services. We identified two overarching themes, professional relations and the regulative framework and current rule and norm system, in the reviewed literature concerning facilitators and hindrances of user involvement in mental health services. By including the interrelated policy concept of ‘active citizenship’ and theoretical concept of ‘epistemic (in)justice’ in the analyses, the results provide foundations for broader exploration and problematization of the policy ideals of what we call ‘epistemic citizenship’ and contemporary practices in Nordic mental health organizations. Our conclusions include suggestions that linking micro-level experiences to organizational macro-level circumstances opens up avenues for further research on service user involvement.

## Introduction and research questions

This paper presents a meta-analysis, drawing exclusively on qualitative research (*n* = 38) published in the period 2017–2022, to contribute fresh findings on contemporary mental health service users’ experiences of received provisions and encounters in the context of Nordic mental health organizations. More precisely, it covers research in Swedish, Norwegian, Danish, and Finnish welfare settings, but not those in Iceland, where service user involvement is less strongly promoted in national policies (1).

It is widely recognized that contemporary notions of individuals with mental illness are often strongly linked to subjects who are usually viewed as different, deviant, and marginalized (2, 3). Stigmatizing notions are embedded in the concept of mental illness that strip stakeholders of their capability and credibility as ‘epistemic subjects’, that is persons who are to be considered credible and reliable sources of knowledge and capable individuals (4, 5). Nonetheless, such theoretically marginalized epistemic subjects’ contributions to knowledge policy and practice—as epistemic citizens—are both valued and sought in the development of high-quality mental health services in Nordic contexts. In this paper, mental health services and organizations are defined as any organizations and institutions that provide help and assistance for people with mental illness, such as primary health care and social service organizations, as well as those providing specialized care requiring referrals (e.g., psychiatric services).

A common feature of the Nordic countries’ contemporary welfare systems is an ideologically driven prioritization and encouragement of *service user involvement* to strengthen service users´ influence on the design and content of received mental health and social services. However, different methods and strategies have been applied in efforts to achieve these goals in the four countries (1).

Service user involvement is related to *active citizenship*, rooted in notions regarding the division and sharing of responsibilities between citizens and the government [*cf.* (6)]. The state is widely regarded as having responsibilities to ensure the welfare of its citizens, while certain responsibilities are ascribed to the individual citizen, such as labor market participation (7, 8), not just in terms of being empowered in the role of being a ‘patient’ or a ‘client’ in a subordinated social position, but also in policy terms of being an *epistemically active citizen* [hereafter an *epistemic citizen*] [*cf.* (9)]. The (pro)active citizen is also regarded as having primary responsibility for making good and healthy choices, for instance in Swedish national *health* policy, which are expected to be based on (or closely aligned with) information and recommendations dispersed by the states’ health organizations [*cf.* (8)]. However, epistemic *capability* is essential in order for citizens to take responsibility for their actions, knowledge acquisition, choices and participation in society [*cf.* (9, 10)]. Likewise, promotion of service user involvement presupposes that service users are capable and valuable sources of knowledge for the establishment of appropriate care regimes and processes for them. Thus, this policy goal has democratic underpinnings. Therefore, an overarching explanation for the prioritization of enhancing service user involvement in Nordic welfare policy is that it is related to the lagging political achievement of epistemic citizenship (choice and voice) in patients’ encounters with welfare state organizations as representative institutions of the state.

In the politicized concept of service users’ involvement, notions of person-centeredness are embedded that refer to the recognition of their expressed personal needs, experiences, and preferences. A prerequisite for embodying such a role as a service user is active involvement. Generally, service user-involving practices are intrinsically underpinned by Nordic welfare policies aligned with notions of empowerment, self-determination, and other positive aspects of service users’ agency (1), but they are also linked with organizational-level development of welfare services’ quality. Such ambition to raise services’ quality is reflected in a commitment to evidence-based practice (EBP), which is stressed in organizational regulations and national-level policies (11–13). EBP refers to scientifically proven and efficient interventions/treatments in social service and healthcare practices. It theoretically rests on three equally important epistemic sources: service users’ experiential knowledge, professional experience and practice, and the best attainable knowledge (14, 15). Hence, service user involvement is theoretically a crucial element of practices that are congruent with the epistemic triad model of EBP (12, 13, 16).

This paper focuses on research addressing service users’ experiences of their encounters with welfare state organizations in Nordic contexts. This was motivated by considerable empirical evidence that although service user involvement is strongly, and ideologically, promoted in these countries, mental health service users often experience disempowering encounters in mental health organizations, and there is low recognition of experiential knowledge (12, 17–23). In sum, this suggests a potential conflict between political ideals and mental health service users’ reality related to their value as epistemic citizens in their encounters with caregivers in ‘professionalized spaces’. If so, use of individuals’ experiential knowledge and involvement in their own care may be strongly promoted directly in policy constructs, and indirectly through the commitment to EBP in welfare services, but much less strongly in practice (14, 20, 23).

Recognition of needs to identify what mental health service users’ experiences consist of (what they are) and meta-analytically *represent* motivated the research presented here. We consider that mapping successful and non-successful service user encounters with welfare actors, as revealed in empirical research, can potentially outline empowering, inclusive, and less inclusive welfare practices and structures as perceived from a stakeholder-perspective.

Against this background we seek to analyze and provide insights into the main facilitators of, and barriers to, mental health user involvement identified in contemporary research on service users’ experienced encounters with professionals in social and mental health services in the Nordic countries. These experiences are analyzed through the interrelated theoretical concepts of epistemic (in)justice and notions of active citizenship. In doing so, we also scrutinize whether and how these encounters correspond to the ambitions of service user involvement and the increased emphasis on high-quality services in these welfare systems.

### User involvement—a work in progress in Nordic welfare

A considerable body of literature and policy texts address the importance of service user involvement and their ability to influence and have an equal voice in decision-making processes, including decisions regarding how assistance and support should be carried out (13, 19, 20, 24–28). More recently, coproduction of services, i.e., service users’ and professionals’ joint involvement in decisions regarding plans and services, has been viewed as a normative ideal in social and mental health services. Coproduced welfare services are also considered to increase autonomy, redistribute power, and improve patients’ recovery (17, 29, 30). Hence, research supports the hypothesis that the active involvement of service users increases the quality of welfare state services [*cf.* (14, 18, 19, 31, 32)].

The organization of mental health services is contextually bound to national traditions and systems, and may vary significantly across nations. However, the Nordic countries have similar systems, with provision of universal support services through taxation of income (33), and where a decentralization of ‘soft’ governance generally addresses municipal or regional responsibility for, and control of, the implementation of public health policy (Fosse and Helgesen, 2019 (34)). According to a recent scoping review by Ineland (1), there is high interest in these countries in the development of methods to enhance service user involvement in practice. Legislation on individual rights, in terms of service user involvement in social and health care, has been passed in all the Nordic countries, but is more limited in Iceland than in Sweden, Norway, Denmark and Finland (1). However, methodological and practical guidelines for approaching notions and practices of service user involvement are under development, from various perspectives, in all the Nordic countries (1, 13, 17, 19, 31, 32).

Practices that are intended to comply with the ideals of service user involvement can be found in different forms and at different levels. At the individual level, one example involves shared decision-making in encounters between service users and professionals. In such practice, professionals actively involve service users in the process of finding treatment options that are deemed most suitable (35). The purpose of such an approach is to empower the service user to ‘take charge’ of important decisions regarding treatment, which is also suggested to promote the continuation of treatment plans and recovery (30).

Another method, which is a quite new and as yet underused organizational approach to promote service user involvement and enrich welfare organizations with service users’ experiential knowledge and perspectives, is to integrate a new occupational category of *peer supporters* in psychiatric services. Peer supporters are former patients with lived experiences of mental illness who have successfully recovered (36, 37). Their main function is to support patients in different welfare contexts by bridging the unequal power distribution between professionals and patients and promoting support aimed at more personal and person-centered care, thus mainly targeting the individual level of service user involvement (36, 38). However, they also function as beacons of self-recognition, empowerment, and hope of recovery for patients with mental illness (39–42). As Argentzell (36) highlights, peer-support workers’ experiential knowledge and perspectives may induce a local recovery-oriented climate in an organization and provide an ethical compass for their colleagues onwards.

Another peer-to-peer approach to strengthen service user involvement and the quality of mental health services, primarily on an organizational level, is to incorporate service user-led monitoring and revisions (43). This involves evaluations of mental health service organizations by various methods, such as interviews with service users and/or surveys underpinned by holistic perspectives (covering multiple aspects of well-being) [*cf.* (44)]. Hermeneutically, the peer-to-peer evaluation of testimonial accounts of received mental health services, together with contributions from the new peer-support occupational role and shared decision-making, may theoretically have substantial potential to counter the unequal distributions of personal resources that are important markers of epistemic (in)justice. More specifically, the deployment of peers’ insider knowledge may reduce inequalities in power relations, through the common ground of lived experiences of being a service user in a relatable social situation with other peers—as a person dependent on the quality and practice of welfare services that are constructed for an intrinsically vulnerable social group.

In the reviewed research, the main thematic incentives to politicize service user involvement in Swedish social and healthcare are underpinned by two democratic notions. First, the promotion of empowerment among stakeholders in order to control their own courses of personal recovery in professional encounters. Second, civic empowerment through redistribution of power to service users *via* user-led evaluations and the development of social and healthcare services where service users control, revise, and suggest improvements in contemporary organizations and services.

### Epistemic injustice versus professional privilege and organization

Epistemic injustice as a theoretical term is not fixed, but rather a spectrum of situations in life where subjects (of various subgroups) are dismissed as equal knowers. The concept can be understood as profoundly associated with a range of normatively deviating social groups lacking credibility in normative daily life contexts.

Drawing on work by Fricker (4, 45), individuals’ *testimonial* injustices and *hermeneutical* injustices are important factors to consider when searching for an understanding of, in this case, service users’ experiences of received social and mental health services that fail or succeed, to meet their needs. Fricker later came to expand her original work on epistemic injustice, recognizing that distributive epistemic injustice refers to information as a type of resource that is systematically and structurally inaccessible for epistemically devalued social groups (45, p. 1318).

Hermeneutical injustice can be described as a (sub-)cultural disadvantage when navigating in particular social contexts, or ‘spaces’, due to the absence of compatible meaning-making resources (4, 45). Individuals’ hermeneutical disadvantages influence their testimonial accounts (i.e., abilities to articulate the ‘right’ questions, personal experiences, and needs). In meetings between service users and professionals, the lack of medicalized knowledge and terminology (in healthcare encounters), legislative rights (in encounters with social services), or the coordination of services may lead to imbalanced epistemic encounters where subjects are dismissed as credible knowers (4, 46).

The general power relations between service users and professionals have been intensively researched. Power relations in an encounter favor the professional through imbalances in both social status and associated ascribed competence, drawing on both hermeneutic and testimonial credibility and authority (46). Further elements of epistemic injustice in meetings between service user and caregivers, besides the hermeneutical and testimonial imbalanced power distribution, have also been noted by researchers. These include informational injustice, as service users may be expected to participate in their own care, but based on the caregivers’ premises, which emphasize the importance of medical knowledge and professional experience [and spaces], and locally situated taken-for-granted routines (5, 46, 47). In such cases, an encounter between a service user and professional is restricted to the service user being a cooperative recipient of, and source of information for, professional knowledge concerning their care. However, the service user is not expected, nor desired, to initiate discussions on alternative treatment options or reject the decisions or assessments of the caregiver (20). This kind of restricted participation leads to what Kurs and Grinshpoon (5) refer to as epistemic silence, a kind of epistemic injustice that occurs in what we define as passive participation, rather than the active participation that is promoted in guidelines or ambitions to enhance service users’ involvement in contemporary policy and practice. Passive participation is hence not a reciprocal encounter, but one that merely demands service users’ presence due to institutional routines and praxis.

Over the years, various studies have highlighted that not being ‘heard’ or ‘understood’ is a common experience among users of social or mental health services [*cf.* (21, 48, 49)]. This calls for enhancement of staff competencies in relational approaches to people with mental illness, which has great recognized importance for high-quality mental health services according to a systematic review by Staniszewska et al. (50). Further, this review concludes that all of 72 included studies (concerning practices and experiences in 16 countries in total) found that appropriate professionals’ practices were crucial for service users to experience high-quality care. However, professional discretion is also bound to the local context of care and, hence, should be considered as a product of the ‘system’—that is, the organizational context.

Demands for efficiency deriving from overarching organizational systems and regulations are prioritized in many rehabilitation contexts (3, 51, 52), which affects several aspects of service users’ agency. Organizational regulations, guidelines, and resources can both weaken the alliance between professionals and service users, and strengthen them, depending on the local organizational context (31, 51, 53).

## Method and data collection

### Qualitative research and meta-analyses

This literature review is based on meta-analyses of qualitative research. It focuses primarily on qualitative empirical research and first-person testimonies as primary data because epistemic knowledge and hermeneutical accounts represent a spectrum of thoughts and situated experiences that are dynamic, complex, and difficult to capture through quantitative methodology, especially concerning mental health and illness (54). Humans interact with their environments, so a deep understanding of their experiences is not easily captured in a reductionist manner, such as that applied in many quantitative methodologies (54–56). In contrast, qualitative methods and methodologies highlight the importance of person-first accounts, thereby emphasizing the epistemological appraisal of lived experiences.

The fundamental goal of qualitative meta-analysis is to provide a comprehensive but concise account of research findings on a focal topic (55, 56). We decided to apply this strategy to search for common themes and patterns in findings of qualitative studies in order to aggregate knowledge regarding service user involvement and epistemic (in)justice for persons with mental illness in their encounters with welfare organizations. However, the form of knowledge production may vary depending on the purpose of a meta-analysis. In some cases the main aim may be to understand conflicting research conclusions or approaches, while in others (as in our review) it may be or to find essential elements that illuminate common denominators of sampled studies (55).

Our analysis and choice of study design are inspired by the work of Levitt (55) and Levitt et al. (57) and the guidelines on methodological integrity provided by the American Psychological Association (APA) for promoting the trustworthiness of the process and results of a meta-analysis. Two overarching principles (with various sub-categories) for the trustworthiness of meta-analytical work are *fidelity* and *utility*. Two key aspects of fidelity are *adequacy* (of studies included in a review to cover the focal topic sufficiently), and *groundedness* (of the analysis and construction of categories in the data). *Utility* refers to the correspondence between the aim and study design, and the study design’s viability in relation to its stated purpose (57). These aspects are addressed in the following section by outlining and justifying the procedures applied in our study’s initial phases.

### Study design

An important aspect of methodological integrity is the umbrella concept of fidelity, which reflects the application of steps in the data selection process that avoid narrowing the rich variety of data under study to a few aspects (57). Accordingly, the first author, in collaboration with the university’s library services, constructed several search strings that included synonyms and other conceptual varieties to increase the probability of finding a generous range of studies concerning users’ experiences of Nordic social and mental healthcare services (see Appendix 1). Then, we searched a range of databases (SocINDEX, APA PsycInfo, Scopus, and PubMed), aiming to include studies rooted in diverse academic disciplines due to the complex life situations and needs of service users. The ‘hits’ were narrowed by using a “peer-review” checkbox, publication date spanning 2017–2022 and the additional criteria of “narrative,” “focus group,” and “interview” in the study designs. As illustrated in the flowchart shown in Appendix 2, the search strategy yielded 860 peer-reviewed studies in total, but despite the search criteria applied quantitative methodologies were used in many of the studies. In addition, some were conducted outside the Nordic countries due to authors having Nordic university affiliations. An additional mechanical search process was performed after importing the publications into Endnote software, using the search terms “narrative,” “focus,” and “interview” to select all the publications containing these terms in their titles or abstracts. In total, 523 abstracts were selected. The first analytical process to include or exclude publications began with reading these abstracts. Papers were excluded if:

1. They addressed populations who did not have a mental illness as their primary diagnosis, but comorbidity (e.g., depression/lowered quality of life as a result of a non-psychiatric diagnosis, such as cancer, epilepsy, or arthritis).
2. They applied quantitative methodology, or qualitative methods with a modest number of quotations from informants (service users), making it difficult to evaluate the groundedness of the authors’ analysis in the presented data.
3. The presented studies were methodological or evaluative, dealing for example with new projects (pilot studies), to maintain the focus of exploring experiential knowledge in previous and existing welfare provisions.
4. The research participants were less than 18 years old. Due to the intrinsically different social and healthcare systems for adolescents, it was not deemed suitable to include a young population in the study design.
5. They were published in 2017 or later, but declared that the presented data were collected before 2015. These were excluded to analyze recent situations and experiences. Studies published in the same timeframe that did not declare in the abstract or main text what year the data were collected were not excluded.
6. The populations under study represented service users with drug abuse issues, and the papers did not focus on needs regarding social and mental health services.
7. They were duplicates of included articles.

After this initial inclusion and exclusion process, 67 publications remained and were subjected to full-text readings, after which 38 peer-reviewed publications were included and further analyzed.

It should be mentioned that despite our Nordic perspective and interest in this study, Iceland was not included in the search strings used, because (as already mentioned) the emphasis on mental health users’ involvement in welfare policy is modest in Iceland compared to Sweden, Norway, Denmark, and Finland. Thus, experiences of service user involvement in the deviating welfare context of Iceland could have potentially compromised the coherence of the review’s findings (1).

### Analysis

Overall, the analyzed dataset was comprehensive and touched upon several perspectives and aspects of mental health service user involvement and (indirectly) just and unjust epistemic encounters. As shown in Appendix 3, although many of the articles related to psychiatric care, overall they covered a great variety of contexts, service provisions, and testimonial accounts of encounters in social and mental healthcare services.

The initial analysis was conducted through a deductive approach. In accordance with directed content analyses (58), we explicitly searched for lived experiences of services and professional encounters. Sections in the articles touching on social networks or experiences of having a particular diagnosis were excluded from the analysis. In articles addressing both service users’ and professionals’ narratives and experiences, only quotations from service users were analyzed. Thus, our analyzed texts consist predominantly of quotations from research participants regarding their own experiences of social or mental health services. However, when relevant to the context, we also included the authors’ discussions and elaborations in the articles’ results sections in our analysis. These passages were checked for relevance against the presented informant quotations to assess the level of abstraction from the primary data. The quotations were also subjected to a coding process, in which we condensed them into several briefly descriptive codes, ranging in length from one word to a short sentence.

Before we started analyzing these codes a user committee comprising individuals with personal experiences of social and mental health services was contacted. They engaged in coproduced elaboration on a random sample of publications (*n* = 7) during a workshop session with the first author. The committee participants read and analyzed the data with an inductive approach. Later, we discussed the main findings the participants identified. These contributions were taken into consideration in the initial stage of our own analytical process, mainly targeting the relational and epistemic injustices in the data and were later confirmed by the authors after analysis of the complete material. The identified codes were reread several times until patterns were recognized and the codes could be organized into two overarching themes: *professional relations* and *the organizational context*. For a more tangible understanding of each theme, the codes under these respective themes were re-read, which resulted in the formation of sub-themes. We subsequently revisited the primary data to ensure that the (sub)themes reflected a valid level of correspondence and abstraction.

## Results

The findings in this paper illuminate diverse experiences of being a service user with a mental illness in contemporary mental health organizations. We have identified two broad themes with recurring subthemes in the 38 analyzed studies. Findings show that narratives of mental health services users in the Nordic countries – negative and positive – and their experiences of participation and (in)justice are related to two explanatory aspects. One consists of the characteristics and quality of professional relations, while the other consists of the regulative framework and current rule and norm system of the services. Therefore, the analysis identifies constituents of service user involvement through individual experiences, relational encounters, and organizational prerequisites. We also examine how these constituents work together to give meaning to service users’ testimonies and a position as citizens of epistemic worth. We argue that these relations represent pivotal aspects of the complexity embedded in defining, discussing, and understanding issues relating to epistemic justice within mental health organizations. Most, if not all, of the papers touched upon mental health patients reporting on their relations with professionals and the professional world. Positive experiences were predominantly connected to confidence and safety in professional encounters and characterized by, for example, continuity, responsiveness to individual needs, and the abilities to build trust, achieve a sense of uniqueness, and be recognized as a ‘person’ rather than a ‘service user’ (59, 60).

Taken together, professional relations between patients and professionals are crucial for service user involvement, empowerment, and consequently, epistemic justice. However, our analysis also suggests that the structural and organizational context strongly influences service users’ intersubjective perceptions and experiences of involvement in various ways when positioned as ‘service users of the welfare state’ (1). In sum, the results section reports findings of positive and enabling, as well as negative and obstructing, experiences and prerequisites for service user involvement and patient-centered care in mental health services in Nordic countries. By doing so, our study adds fresh findings regarding evidence-based welfare services and the growing body of research on what attenuate and undermine mental health patients as epistemic citizens. As shown in Table 1, we summarize our main findings by differentiating between two overarching themes and several subthemes. In the following text we provide more detailed information (with empirical evidence) on how these themes and sub-themes differentiate positive from negative user experiences within mental health provisional encounters.

**Table 1:** Overview of themes and sub-themes.

Themes	Sub-themes
Professional relations	Physical and emotional accessibility and availability Autonomy and safety	Responsiveness to individual needs and preferences Empowerment and reciprocity
Organizational context	Information and knowledge distribution Continuity and organizational fit Co-productive working processes Ideology

### Narratives and experiences associated with professional relations

One main finding of this scoping review is that numerous studies emphasize the importance of professional encounters and how welfare state systems – through their professionals – respond to mental health service users. This is crucial as the ability to establish empathetic relationships is commonly recognized as a keystone for quality in human service organizations (61). From a service user perspective, the quality of professional relations is also crucial for the development of trust and willingness to open up (60, 62, 63). The importance of relational aspects in mental health services is illustrated by a study of residents in supported housing for people with mental illness (64). The results show that easy access to professionals reduced patients’ frequencies of hospitalizations, which the cited authors regarded as an indication that the availability of significant others to help them cope at difficult times improved their self-regulation experiences (64, p. 69).

A recurrent theme in our findings is that service users’ experiential knowledge is valued, recognized, and called for, although the review does not provide clear information on the *extent* that service users, as epistemic sources, are listened to and involved in actual decision-making and work processes within the Nordic mental health sector. In a study of recovery-oriented intersectoral care in mental health, Jørgensen et al. (65) show that although health professionals acknowledge the value of involving mental health service users and relatives, and call for their opinions, care decisions are largely made paternalistically, and such voices are ignored. Instead, our review indicates that encounters and communication patterns characterized by an absence of stigma and imposition seem to be, in themselves, an aspect of epistemic justice [*cf.* (59, 66)]. In the following text, we present more detailed findings according to the themes and sub-themes outlined in Table 1 on service user narratives and experiences of influence, voice, relations and organizational settings.

### Physical and emotional accessibility

The ability to encounter physically and emotionally accessible professionals is by far the most frequently reported aspect of professional relations and most explicitly differentiates positive from negative user experiences of the mental health sector. Although some positive examples are reported in the reviewed studies, these accounts were essentially challenged or overshadowed by experiences and narratives of a negative nature (62, 67–71). Other papers also report on ambitions and preferences of patients, emphasizing that they want to be recognized, listened to, and acknowledged as capable persons with valuable knowledge about their own current life situations, i.e., they addressed desires for epistemic recognition [*cf.* (4, 46)] and not to be ignored, dismissed, or condescendingly treated.

Moreover, inaccessible professionals are important elements of negative experiences, as shown for example by Pelto-Piri et al. (70) and Brännström et al. (72). This indicates that a lack of communicative staff, non-engaged professionals, inadequate meetings, and one-sided interactions cause feelings of being ignored and neglected by staff working on ‘autopilot’ (59, p. 544). Our review, however, shows that confidence, trust, and sustainable relations with professionals can make them feel ‘unique’, recognized (59, p. 543) and ‘safe’ (68, p. 596), which are recurrent themes of informants’ descriptions. The following extracts are typical examples:

The participants described experiences of feeling listened to; professionals were described as being “focused” and “present” and they “listened while still maintaining their professionalism” (59, p. 542).

It makes me happy when the occupational therapist asks if we should bake a cake or go for a walk [in the meadows near the mental health center]. I can live on this kind of experience for weeks (68, p. 596).

For all participants, descriptions of their relationships with ward staff permeated throughout the six components [under study] … underlining the centrality of developing personal relationships between patients and staff in creating a therapeutic ward atmosphere … (71, p. 344).

Another important finding is that, regardless of scale and specialization, organizations that value user-involvement and successfully implement it in their praxis (with utilization of users’ experiential knowledge) have positive effects on service users’ recovery (63, 73–75). What we depict as positive and reciprocal encounters also seem to enhance trust in the welfare system (60, 62, 76), motivation to use services (69, 77, 78), and users’ control in their encounters with the professional world (63). In contrast, many of the reviewed articles indicate that patients experiencing professional relations as malfunctioning, distrusting and stigmatizing lead to negative feelings of being personally insignificant, worthless or de-humanized [e.g., (68, 76, 79, 80)]. Thus, our analysis suggests that service users’ subjecthood plays a key role, due to strong associations between self-perceptions of being socially valued and positioned as a fellow human being who is worthy of epistemic recognition, which is manifested in the presence of reciprocal engagements with professionals.

### Autonomy and safety

Our results show that the quality of mental health patients’ relations with the professional world contributes considerably to their feelings of autonomy, safety, and security. In sum, the results stress the importance of creating a safe institutional environment and actively involving service users in their care by creating an open, confident, and safe place for dialogue (62, 81). Some studies found indications of negative outcomes (63, 82) related to unconducive group compositions or the absence of physical encounters, which were perceived as impersonal and barriers to trust and safety (62). The following excerpts are illustrative examples of how dismissing, non-engaged, and non-communicative professionals can contribute to patients’ isolation and feelings that they are unsafe and bear responsibilities beyond what should be expected of an inpatient:

We [inpatients] have a lot of people who go through periods of feeling very bad here … and we bear the responsibility for whether they will live until the next day when they indicate they have suicidal thoughts or have attempted suicide and the like … We don’t really know if we’re able to deal with this (70, p. 6).

The participants do not experience a focus on their recovery process across sectors, and the medical treatment paradigm undermines their own perspectives on life. “The doctor filled me with medication, and I slept all the time. I said to him: Tell me, do you want me to sleep my life away? Yes, the doctor said” (65, p. 9).

A number of studies reported positive narratives and experiences (60, 66, 73, 74, 81, 83, 84). For instance, Björkvik et al. (73, p. 32) showed that service users’ propensity to use dental health services was strongly related to their feelings of safety and their perceptions that their dentists understood and respected them. The delicate nature of interpersonal relations and their importance for users’ feelings of safety and a sense of control are also evident in the following excerpts, indicating the significance of the help given and engagement shown by professionals:

Without ES [psychiatric nurse], I would never have been here today. She called me an hour before picking me up and came together with me (73, p. 32).

The group members described the feeling of being safe first and foremost as a feeling of trust and acceptance. These men had their triggers in the interpersonal field/ … /they emphasized confidentiality in the group more than their physical security [following an outburst, author remark] (82, p. 8).

These sub-themes thus illustrate differentiated perceptions and experiences based on the quality of interpersonal relations and professional accountability within mental health welfare contexts. Safe, confirmatory, and non-coercive contexts are described as prerequisites for reciprocal and respectful professional relations, which are fundamental elements of not only service users’ involvement and sense of autonomy, but also their hermeneutical and testimonial credibility [*cf.* (4)]. Consequently, qualitative professional relations also have high potential for identity-building (78) and improvements in both self-esteem and recovery processes (73, 75, 77, 82). In contrast, non-successful encounters, characterized by limited reciprocity and service user influence, safety, and autonomy, increase risks for shame, dehumanization (being reduced primarily to a ‘service user’), fears of airing one’s opinions and sanctions, and reduced opportunities to foresee future steps of a given recovery or rehabilitation process [e.g., (65, 85–87)]. This is a significant obstacle for implementation of the fundamental ideals of service user involvement and epistemic citizenship in the Nordic countries. There are high risks that welfare contexts within them may not deliver provisions permeated by empowerment, coproduction and diverse forms of recovery if relations within them induce such negative and reductionist effects on personal autonomy and voice.

### Responsiveness to individual needs and preferences

The third subtheme of how professional relations seem to differentiate positive from negative service user narratives is individual recognition, i.e., professionals’ ability to respond to individuals’ needs and preferences, in line with Nordic policy aims to enhance service user involvement [*cf.* (13, 23, 28)]. Jones et al. (79) and Hagen et al. (60) present negative and positive service users’ narratives and experiences regarding their encounters with the professional world. The significance of professionals’ responsiveness to individual needs is evident in the following two quotations of participants in the study by Jones et al. (79):

[Professionals] need to hear me and be able to understand … I have PTSD and people [professionals] who do not know what PTSD is, cannot understand why I am like I am, nor can I get help then from someone who does not know what problems I have.

I have had a lot of psychologists, contacts, and similar, but none of them have worked because they have followed these routines that they have, rather than looking outside the box, but then I got someone who listened to what I said, really … saw me as a person and listened to what I had to say … it was a huge help.

In their study on former suicidal inpatients, Hagen et al. (60) also address service user experiences related to professionals’ responsiveness to users’ testimonial accounts and individualized support. They suggest that to improve the quality of professional encounters, and provide more individualized care, professionals need to use more extensively not only their professional but also their personal qualities and act as empathetic fellow human beings. Some articles report patients feeling that their perspectives and experiences were overlooked, dismissed, or overshadowed by professionals, guided primarily by ideology or routines, potentially leading to neglect of their individual wishes and invalidation of their lived experiential knowledge (72, 88). In contrast, recognition of and responsiveness to individuals’ needs and preferences can potentially enhance patients’ recovery processes, as shown for example by a study of people who had common mental disorders and had experienced sickness absence (89, p. 9):

One factor that emerged from the participants’ experiences of professional support was the importance of being listened to and that someone believed in their story. This mutual respect was vital for achieving recovery.

Sunnqvist et al. (90) also touch on the importance of respectful and responsive meetings with people with mental illness in their study on prehospital emergency psychiatric units. Failure to provide such meetings may have negative consequences that leave patients feeling reluctant to seek care, in line with previous findings. In one example of their importance, a professional took time to talk calmly and respectfully with ‘Patient 3’, creating a trustful alliance, which made the patient feel safe: “So if it had not been for him, I would probably still have sat in my apartment … refusing to leave …” (90, 259). Coproduction of services in such cases is represented in terms of active agency (voice) and reciprocity and alliance (reciprocal relations) between service users and professionals. These are prerequisites for any form of coproduction in welfare contexts [*cf.* (19, 26, 27, 29)] and crucial for active participation in services.

### Empowerment and reciprocity

The empowering potential and measures of reciprocity constitute the fourth subtheme of professional relations. Relations that acknowledge and promote involvement of service users and their experiential knowledge in daily routines are associated with positive outcomes, while opposite kinds increase risks for non-participation, us-them dichotomies, and lack of choices for service users accompanied by other disempowering practices, as shown by various authors [e.g., (68, 70, 76, 80, 85)].

Eldal et al. (76) highlight a recurrent theme in our review—the challenging service user position of engaging in professionalized spaces, due to the unequal power-distribution—that, at times, caused situations where service users’ subjecthood was marginalized. One patient framed this as “scary” and “a risk” in their role as a service user (76, p. 796). Pelto-Piri et al. (70) also address relational aspects in terms of us-them narratives and provide vivid descriptions of service user narratives of being a burden or disturbance to professionals when asking questions or wanting something demanding consent, which causes disempowerment and increases patients’ fear of conflict.

Some researchers have indicated ways that professional relations may also potentially help to re-distribute power, increase reciprocity and enhance patients’ empowerment. Although it may be challenging, Møllerhøj and Stølan (68) argue that even the smallest professional initiatives may be important for motivation and meaning:

The informants are very well aware of the power relations at stake, and the fact that the responsible consultant decides at the end of the day. However, the feeling and experience of some sort of negotiation and shared decision-making are important to patients [adjusting medical treatment] … (68, p. 596).

In terms of providing opportunities for involvement and positions as epistemic citizens, the review also reveals that relations reflect signs of genuine interest and recognition of service users as human beings, mutual trust, honesty, and reciprocity (78, 91–93). They also help to avoid feelings of shame, stigma, and anxiety (62, 73, 95) and increase individuals’ sense of power and control (63, 77). In sum, the findings presented in this section provide nuances of the commonly held view of how public organizations—through *reciprocal* face-to-face encounters between professionals and service users—acknowledge people with mental illness, which is a pivotal aspect of service quality, and hence epistemically just encounters in mental health services. The reviewed articles indicate that reciprocity occurs in encounters where the social position and subjecthood of mental health service users are not epistemically challenged by, nor dismissed in, professionalized spaces and authority [*cf.* (4, 5, 46)] and where their epistemic citizenship can both be practiced and valued.

## Narratives and experiences associated with organizational settings

In line with previous research, it is clear from our analysis that social and mental health organizations pose challenges when interacting with service users and patients with specific needs and preferences, due to their legal, moral, and institutional frameworks [*cf.* (96)]. A major reason for this is that institutional frameworks provide guiding principles for actions and engagement with individuals positioned as service users of the welfare state [*cf.* (3, 49, 51, 52)] that may exacerbate rather than ease difficulties in their recognition as human beings with individual biographical, cultural, and illness-related histories (92). The institutional frameworks of mental health organizations also serve to distribute power and influence among various organizational actors, which participating patients highlighted in the reviewed studies, as illustrated by the following conclusion of Møllerhøj and Stølan (68):

Participants were aware of the fact that there was a care hierarchy in which the patient was at the bottom. They described powerlessness in relation to staff and there were some descriptions of oppressive behavior from the staff.

Although professional relations and organizational contexts are conceptually different, they are also intimately intertwined. Financial restraints, understaffing and paucity of local guidelines for patient-professional interactions or collaboration in inter-organizational teams will most likely negatively affect professional relations with service users. Thus, the physical absence of nurses in inpatient settings and interrupted service user-professional conversations, for example, have been treated as organizational, rather than relational, factors. Our findings suggest that being given sufficient information and the coordination of support structures contribute positively to service users’ experiences, while a lack of coordinated and collocated services negatively affect their motivation and willingness to contribute to their recovery process (67). This results section reports findings that, from our theoretical and analytical standpoints, represent how service users experience their involvement and epistemic citizenship (participation, agency, and navigation) in mental health services and how these narratives are associated with the organizational context.

### Information and knowledge claims

The ways that organizational contexts promote or limit service user involvement initiatives, as well as service users’ experiences of agency, are most clearly related to issues concerning information, the forms of knowledge that are valued and acknowledged, and how the valuation and acknowledgement are manifested (81, 90, 93). As shown by the following quotation from a participant in a study on patients’ experiences of caring encounters with a psychiatric mobile emergency response team (81, p. 445), adequate information has a preventive function and instills trust and safety in patients:

They told me when to take the sleeping medicine … to wait until I was in bed; in that way, I would reduce the risk of falling … they also told me to contact the ordinary (psychiatric) mobile team or them before harming myself.

This quotation clearly shows that information in the form of self-care advice aided the handling of a situation before contact was resumed with regular caregivers. However, the literature commonly reported mental health patients describing ongoing or previous experiences with welfare services in terms of resistance and mistrust due to limited knowledge about the welfare system. This touches on important aspects of initiatives for service user involvement, as information has empowering potential for patients in mental health services:

They [professionals] have become better and better at helping me because I am getting better and better at knowing what I am entitled to or not! (86, p. 195)

If service users cannot acquire information on entitlements in welfare services from professionals they must acquire it from other sources. Such lack of information can cause feelings of obscurity and insecurity in the ‘helping alliance’, with ‘help’ being perceived as deceptive. Such epistemic (hermeneutic) injustice due to the lack of information can arouse strong feelings in service users, of their lack of knowledge being acted upon by professionals, rather than being provided with answers and information [*cf.* (45)]. Several reviewed articles identified examples of negative effects and experiences due to insufficient or inadequate information and situations, when service users’ experiential knowledge was neglected (66, 80, 85, 86, 91) or service users found it challenging to share, connect, or engage in genuine negotiations with professionals during treatment (83). Our results indicate that well-informed service users are both more motivated and hermeneutically better equipped to raise awareness of individual needs and preferences in professionalized spaces. Being well-informed also seems to empower users of mental health services as active citizens, challenging and resisting what are considered coercive and unethical practices [*cf.* (93)]. In contrast, the lack of information or patients not receiving information at all decreases motivation and strengthens the individual’s role as a ‘service user’. This reinforces the us-them dichotomy between professionals and service users due to practices that strengthen the difference in epistemic (hermeneutic and testimonial) authority between the parties:

Some patients had excluded themselves from the planning and, due to lack of motivation or confidence, found it easier to adopt an outsider’s role in their own care/ … /the participants agreed that patients need sufficient information on medication to participate, but that in practice, patient counseling is insufficient and unsystematic (85, p. 234).

Work by some authors, e.g., Roos et al. (75), showcases how a lack of information compromised patients’ preparation for rehabilitation, causing them to constantly repeat themselves, which negatively affected their motivation and recovery processes (80). This is congruent with findings of previous research [*cf.* (35, 50)] addressing issues related to what Kurs and Grinshpoon (5) refer to as ‘epistemic silencing’. In such cases, organizational routines or structures cause hermeneutical and testimonial injustice due to a lack of information and proper support, leaving individuals to opt-out from their own care and recovery process. Together with unclear role responsibilities and ambiguous rules and routines, a lack of knowledge and failure to integrate experiential knowledge into the work process have also been identified as major obstacles to service user involvement (86, p. 194). This confirms recent findings regarding hindrances for the realization of epistemic citizenship in mental health practices [*cf.* (17, 19, 31, 32)].

### Continuity and organizational fit

A recurrent theme in the research participants’ descriptions is a low degree of continuity and structure in their contact with mental health services, causing challenges in managing their mental illness. Lockersten et al. (80, p. 6) provide an illustrative example, of organizational misfit causing fear and halting of the recovery process for young adults with eating disorders:

When treated in in-patient care, they were admitted with other patients who had been ill for a long time. These factors influenced the participants’ hope for their recovery in the future. “I was admitted with patients that had been ill longer than I had been living.”

Another example is provided by Stige et al. (95), addressing the link between time and psychotherapy. They conclude that imposing a strict time restriction might “… interrupt and end fruitful therapeutic processes prematurely, forcing clients to seek treatment elsewhere and start all over again with a new therapist—a strenuous and time-consuming exercise.” Other studies show that repeated changes in staff, schedules, methods, etc. can complicate patients’ contact with professionalized spaces. The following quotation from a patient in an outpatient clinic clearly shows that constant changes can results in different professionals making different assessments, decisions, or (rehabilitation) plans, allowing little involvement and causing both frustration and misunderstandings:

Things that may be small, like wanting to get in touch with your psychologist, when it doesn’t work, it adds a little to my heap of things. /—/There have been so many changes in my contact with psychiatry, which has been difficult in several ways, it hasn’t been difficult just because of the way I feel, but it has also been difficult as a result of the way I have been treated and not taken seriously (72, p. 6).

Patients have also reported that such changes have sometimes led them to become over-responsible for their own treatments, which often made them feel less confident. Andersson et al. (89) found that such responsibility “… weighed heavy on them [patients] and was described as a source of worry over, for example, not being able to give the correct health information or suggest the most relevant intervention to the physician.”

At least in part, patients’ experiences of organizations failing to understand and acknowledge individual needs and preferences seem to correlate with insufficient communication channels. Eckerström et al. (66) also noted the disruptive consequences of employee turnover, which complicates the distribution of knowledge and ability to establish empathetic and sustainable interpersonal relations. Consequences of such factors, expressed by some patients, may include feelings of being “an object” or a sense of no longer feeling like a human (68, 80).

### Coproduction in working processes

This sub-theme concerns service users’ opportunities to engage directly with professionals, which we regard as organizational and structural factors [*cf.* (1)], and strongly influence their narratives and experiences of being recognized as an epistemic citizen. These findings are important as service user involvement and successful coproduction of services are considered crucial for the quality development of mental health services provided in the Nordic countries [*cf.* (1, 43)]. Our data suggest that the prevalence of coproduction in working processes, which may differ widely in scope, affects service users’ overall experiences in their encounters in professionalized spaces (94).

Participants in the reviewed studies mainly reported negative experiences of shared decision-making or coproduction opportunities, emphasizing that they were inadequate or non-existent. Lindberg et al. (92, p. 640) reported patients’ experiences of being “infantilized and patronized” by professionals [see also (86, p. 197)], which may have profound negative impacts on their self-esteem. Professionals have also been portrayed as homebound, mostly occupied in their offices, and as distant from patients, causing feelings of being “on the other side looking in but not seen” (63, p. 182).

However, there is considerable evidence in the reviewed literature that service users have mixed feelings and experiences (83, 92). Some articles suggest that patients may feel accepted, protected, and safe (as shown in previous sections), but at the same time miss having direct contact with professionals and experience limited choice (voice) and influence (63). These perceptions and feelings highlight vital, but contrasting, aspects of service user involvement initiatives and opportunities for users to draw on their experiential knowledge to add important insights for mental health organizations’ praxis. One participant in the study by Derblom et al. (59) highlighted the potential dilemma involved:

When you [staff] listen to me and process what I say, then you are the expert and I listen to you … because I trust that you are the expert; you know best and also want the best for me.

This quotation emphasizes the importance of knowing, understanding, and ‘seeing’ each individual for the ability to provide individualized assistance and support. Lofthus et al. (67) show that an apparent advantage of participating in an ACT program is that it helps prescription of the correct medication and its adjustment to provide the correct dosage. At the same time, individuals’ rights might be neglected or even pushed aside due to the medication. However, Lofthus et al. (67) conclude that patients experiencing the most restrictions are the ones with the highest reported recovery. These results provide important nuances for ongoing discussions of service user involvement and epistemic justice within the discourse on mental health services.

### Ideology

The fourth and final aspect of the relationships between the organizational context of mental health service provision and service users’ narratives and experiences involves ideology and taken-for-granted assumptions about what is ‘desirable’ and ‘appropriate’ when providing assistance and support to people suffering from mental illness (80, 88, 95). One way in which ideologies are put into practice is through working methods. Røberg et al. (82) provide an illustrative example of how specific (psychoeducational) interventions, in combination with an accepting group atmosphere, can increase self-acceptance and reduce shame and stigma among (male) patients. However, when welfare organizations cannot individualize policy intentions, such interventions may have negative effects. One example is the study by Stige et al. (95), which illustrates how psychotherapy with a predetermined timeframe for recovery was experienced as a burden for many patients (88). One apparent aspect of ‘ideology’ and how it relates to research participants’ experiences and narratives is associated with the ideological characteristics of service provision and a tendency to agglomerate humans with different backgrounds and needs into an impersonal category of ‘service users’. The recovery process is then no longer individualized, but treated as a calculated cost-efficient intervention that service users’ are responsibilized to manage [*cf.* (8)]. Participants in the study by Lockersten et al. (80, p. 6) provided further examples of the logic of welfare state organizations:

With the experienced alteration from being treated as an individual to being treated as an illness, the participants often felt like an object during the transition, dependent wholly on a relationship that was restricted more to the registration of symptoms and less to what they felt would help them. They verbalized a sense of no longer feeling like a human.

The transition mentioned here was from a children’s psychiatry clinic to an adult psychiatry clinic. This was a major change for young adults with eating disorders, who did not feel ready or willing to change the professional contacts who they had confidence in and had known them for a long time. Such transitions that are mandatory due to organizational structures pose risks for losses of confidence and trust in the system, as well as promoting fear of the adult (impersonal) world of psychiatry. Summing up, our findings show the importance of active collaboration within the welfare sector so that patients have the benefit of continuity and experience strong, transparent links and connections between different resources and mental health professionals [*cf.* (80, 82, 88, 95)].

## Discussion

This study focuses on facilitators of, and barriers hindering, service user involvement in social and mental health services in the Nordic countries, which have been analyzed from perspectives of epistemic (in)justice and active citizenship (4, 5, 45). Drawing on a meta-analysis of contemporary research, our findings add new insights to the reciprocity between individual experiences and overarching ambitions for high-quality services expressed in each of the four included Nordic countries (11, 12, 19, 31, 32, 43, 97). They also extend insights by offering empirical evidence regarding two key explanatory factors that help to differentiate between service user experiences: professional relations and the organizational context. Although they are conceptually different, these factors are also closely intertwined. Particularly in financially restrained and understaffed organizations, vague guidelines on patient-professional interaction and/or collaboration in inter-organizational teams will most likely negatively affect professional relations with service users [*cf.* (3, 50–52)].

In line with a meta-analysis by Staniszewska et al. (50), a main conclusion of this study is that professional relations are prominent features of service users’ narratives. Knowledge of these encounters’ quality is crucial for understanding how individuals in the social and mental health sector experience help and support received from the perspective of being an epistemic citizen, and to what extent they are valued as capable individuals with epistemic worth. As an illustrative example, our findings show that the distribution of sufficient information, and successful coordination of support services, positively contribute to service user experiences, while a lack of coordinated and collocated services negatively affect professional discretion and, consequently, individuals’ motivation, capacities, and willingness to contribute to their own recovery process [*cf.* (35)]. An interpretation is that individuals should be enabled to use their epistemic citizenship, for example by receiving information attuned with their hermeneutical resources, and thus enabled to take appropriate action in their current situation, like other (active) citizens. On a personal level this would also validate recognition of their epistemic agency. In addition, empowering and accessible environments—physically and emotionally—or the lack of them, seem to have a major impact on individual experiences of received services in highly professionalized spaces such as those in mental health organizations. By far the most frequently reported individual experiences related to this theme concerned the environmental barriers and facilitators for empowering and accessible care. However, it is important to note that positive accounts were strongly overshadowed by negative storylines (62, 67–71), as also shown in previous research.

Another important insight is that service users’ sense of safety and trust seems to increase when their encounters take place in institutional environments where they experience personal sensitivity and engage in dialogue with professionals (62, 81). This is consistent with another important finding regarding the theme of professional relations; experiential knowledge among professionals seems to be valued, recognized, and/or requested, both implicitly and explicitly, by service users [*cf.* (36, 42)]. Consequently, we consider peer-support an important area for further empirical research. However, the review provides no clear evidence about if (and if so, how and to what extent) experiential knowledge is recognized and applied in day-to-day practice within different welfare organizations. These findings are important as professional relations play key roles in the realization (or failure to realize) the empowerment of service users through their involvement, and consequently epistemic justice. Moreover, the ability to establish and maintain empathetic relationships is commonly recognized as crucial for the establishment and maintenance of high-quality provisions in human service organizations (61). Paradoxically, according to both our analysis and previous research, this ability is lacking in many respects for citizens who need it most. The deficiencies seem to be due not only to a lack of quality in terms of activities or low frequencies of practices involving service users, but also to a lack of fundamental understanding of the critical needs of individuals with mental illness, not as patients, but as human beings. Hence we encourage empirical research attention to the slowly growing approach of engaging peer-support workers in Nordic mental health organizations.

Another conclusion is that service users’ experiences of their encounters with professionals and the professional world seem to be closely linked to the organizational context. Our findings suggest that both professional and organizational aspects are important explanatory aspects to differentiate between positive/facilitating and negative/obstructing experiences of involvement. We conclude that the legal, moral, and institutional frameworks of mental health organizations [*cf.* (96)] seem to pose challenges for engaging with individual needs and preferences. Normative ideals regarding service user involvement and ambitions to equalize epistemic power between service users and professionals are strongly associated with the active citizen discourse in the Nordic countries. Against this backdrop, our findings provide new insights that may contribute to ongoing discussions on guiding principles for (professional) action and approaches when engaging with epistemic citizens positioned as service users of the welfare state. They strongly suggest that the ability to understand individuals’ experiences of their engagements with mental health organizations should be regarded as an institutional element (linked to the rules, norms, and ‘taken-for-granted’ ideas) of these organizations (1, 25, 43, 50). The findings are also connected to the ongoing trend of including working models of service user involvement in quality-enhancing frameworks for practice [*cf.* (11–13, 18, 22)].

Linking micro-level experiences to organizational macro-level circumstances opens up avenues for further research related to epistemic (in)justice and service user involvement (8, 96, 98). To what extent do institutional contexts aid or obstruct recovery processes, well-being, and agency for mental health service users? How do mental health organizations’ rule and norm systems accentuate, conceal, or mystify important ethical aspects of service provision relating to epistemic justice, service user involvement, distribution of power, and taken-for-granted assumptions or perceptions of ‘service users’ and ‘professionals’? Addressing such research questions is important as their answers provide important insights into the moral and epistemic status of people with mental illness as active citizens in the Nordic progressive policy contexts and societies of today. Ultimately they also raise prospects for realizing service user involvement and epistemic citizenship among individuals who need mental health services in the Nordic countries.

When interpreting the results, some limitations should be kept in mind. First, due to the exclusion criteria in the study design we have not considered quantitative measures and findings, which might have added further nuances to our results. Neither have we included parents’ or partners’ experiences of active involvement in service users’ care, which would have added important insights for our analysis, partly because they may provide at least partial channels for the most silent voices, which are often the ones we most need to hear. We should also note some strengths of the study. One is the triangulation in the analysis that was conducted with the local service user committee for collaborative work on a random sample of studies. This was an important contribution that enriched the analytical process with their lived experience and expertise. Moreover, the great heterogeneity of mental health service user groups and organizational contexts that were covered in the included studies probably provided a quite comprehensive and clear picture of contemporary practices and the barriers to and facilitators of service user involvement and epistemic citizenship in Nordic mental health organizations.

### Conclusive remarks and recommendations for practice

In conclusion, this study provides empirical evidence of how ideological, professional and organizational factors may synergistically or antagonistically facilitate and/or constrain the ability of people with mental illness to act as equal epistemic citizens in professionalized spaces (1, 35). Our results show that resources required to empower service users’ agency, i.e., the ability to comprehend and navigate within complex and sectorial mental health systems to obtain necessary support [*cf.* (4, 45)], are intrinsically connected to structural matters. The results indicate that possibilities for individual service users’ to navigate as epistemic citizens are still rather scarce in Nordic mental health services, despite the ambitions to promote active citizenship and user involvement in Nordic policy and practice. These possibilities seem to be heavily constrained by structural aspects, i.e., ideological, attitudinal, and regulatory structures and routines, that must change to enable welfare organizations to provide fruitful and epistemically just relational encounters and support. Soft governance of mental health services in the Nordic countries enables the emergence of diverse locally situated strategies and hence implementation of varying methods and priorities in welfare organizations. It may be time for more stringent policy guidelines, and governance, for addressing mental health issues, as the stakeholders are still facing hardships in modern mental health services after decades of maltreatment and institutionalization. On an organizational level, clear guidelines on active service user involvement strategies should be incorporated followed by staff-education on citizen-inclusive ideologies instead of outdated mental patient-ideologies that belong in the era of institutionalization.

## Author contributions

FN performed the initial search-process in the databases and is responsible for the inclusion- and exclusion-process of records in this study. FN and JI worked joint venture throughout the manuscript. All authors contributed to the article and approved the submitted version.

## Funding

Open access funding provided by Umeå University. This work was supported by the Swedish Research Council for Health, Working Life and Welfare under (Grant number: 2021-01427) and by the Department of Social Work, Umeå university.

## Conflict of interest

The authors declare that the research was conducted in the absence of any commercial or financial relationships that could be construed as a potential conflict of interest.

## Publisher’s note

All claims expressed in this article are solely those of the authors and do not necessarily represent those of their affiliated organizations, or those of the publisher, the editors and the reviewers. Any product that may be evaluated in this article, or claim that may be made by its manufacturer, is not guaranteed or endorsed by the publisher.
