# Supplementary material for: Epistemic citizenship under structural siege: a meta-analysis drawing on 544 voices of service user experiences in Nordic mental health services
Source: Front Psychiatry. 2023 Jun 2;14:1156835. doi: 10.3389/fpsyt.2023.1156835 (PMC10272743; doi:10.3389/fpsyt.2023.1156835)
Supplement: Supplementary file 1 [file Data_Sheet_1.docx]

**Appendix 1**

Search-strings used in the database searches:

"Patient perceptions" OR "Client perception" OR "user perception" OR "patient reflections" OR "users reflections" OR "clients reflections" OR "patient satisfaction" OR "client satisfaction" OR "user satisfaction" OR "user-experiences" OR "user experiences" OR "patient experiences" OR “client experiences” OR "user perspective" OR "user-perspective" OR "patient perspective" OR "user knowledge" OR "patient knowledge"

AND "mental health" OR "mental illness" OR "mental disorder" OR "psychiatric illness” OR “psychiatric health” OR “psychiatric disorder” OR "psychiatric impairment" OR "mental impairment" OR "psychological impairment"

AND "service-provision" OR "social intervention" OR "welfare service" OR "welfare provisions" OR "social care" OR "social provision" OR "social support" OR "welfare support" OR "psychosocial care" OR "aftercare" OR "health care" OR "psychiatric care" "mental health services" OR "social services" OR "community health services"

AND “Sweden” OR “Swedish” OR “Norway” OR “Norwegian” OR “Denmark” OR “Danish” OR “Finland” OR “Finnish” OR “Scandinavia” OR “Nordic”.
