# Supplementary figures and images for: Epistemic citizenship under structural siege: a meta-analysis drawing on 544 voices of service user experiences in Nordic mental health services
Source: Front Psychiatry. 2023 Jun 2;14:1156835. doi: 10.3389/fpsyt.2023.1156835 (PMC10272743; doi:10.3389/fpsyt.2023.1156835)

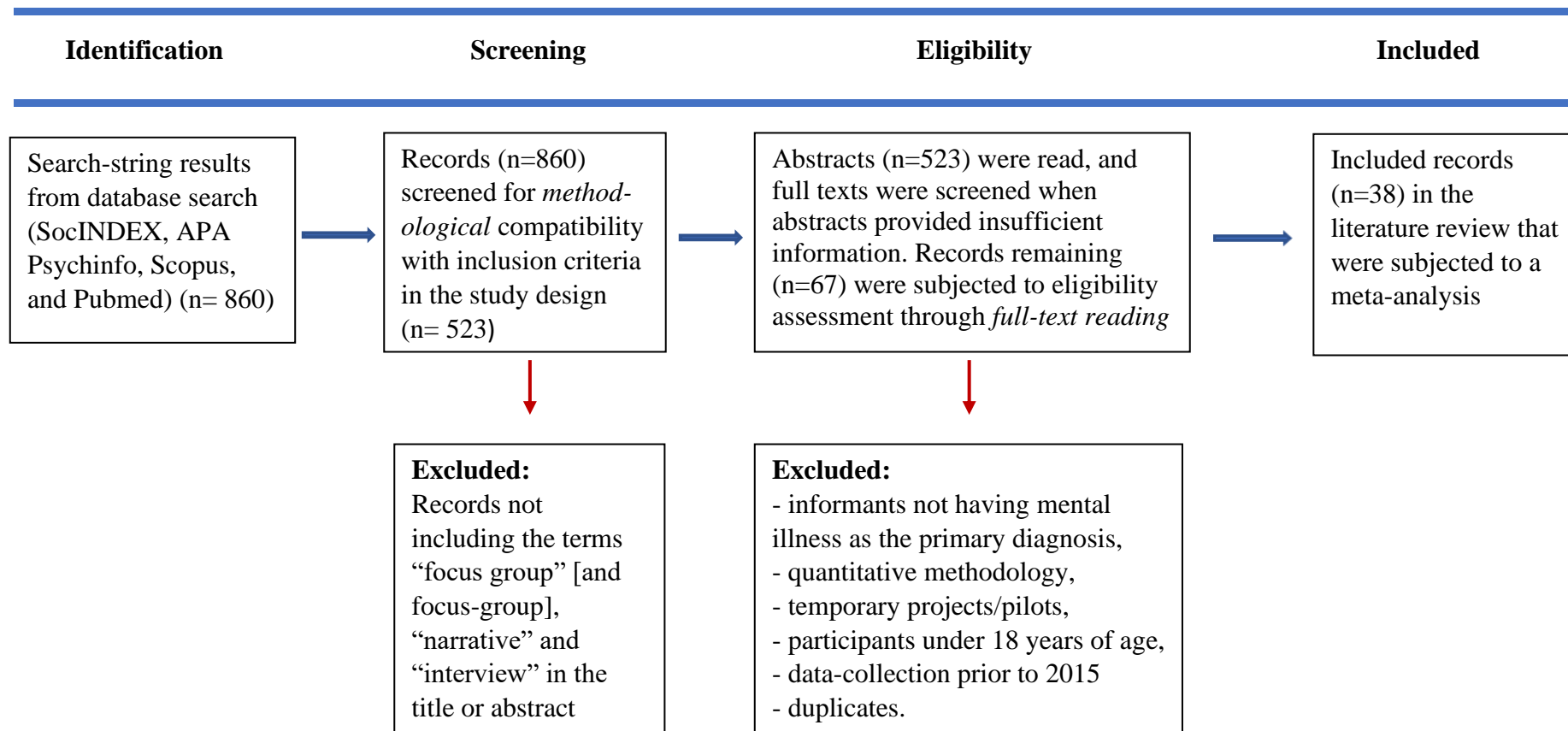

Supplement: Supplementary file 2 [file Data_Sheet_2.pdf]
