# Supplementary material for: Epistemic citizenship under structural siege: a meta-analysis drawing on 544 voices of service user experiences in Nordic mental health services
Source: Front Psychiatry. 2023 Jun 2;14:1156835. doi: 10.3389/fpsyt.2023.1156835 (PMC10272743; doi:10.3389/fpsyt.2023.1156835)
Supplement: Supplementary file 3 [file Data_Sheet_3.docx]

| Authors | Year | Title | Research area | Data Collection | Service users (*n*) |
| --- | --- | --- | --- | --- | --- |
| Andersson, C., Jakobsson, A., Priebe, G., Elf, M., Fornazar, R. and Hensing, G. | 2022 | Capability to make well-founded decisions: an interview study of people with experience of sickness absence who have common mental disorders | Decision making during sickness- and rehabilitation processes. | Semi-structured interviews | 11 |
| Bjorkvik, J., Quintero, H. D. P., Vika, M. E., Nielsen, G. H. and Virtanen I. J. | 2022 | Barriers and facilitators for dental care among patients with severe or long-term mental illness | Dental care | Semi-structured interviews | 51 |
| Brännström, E., Strand, J. & Sand, P. | 2018 | A patient perspective on recurrent or prolonged contact with psychiatric inpatient care for affective disorder | Psychiatric inpatient care | Semi-structured interviews | 10 |
| Derblom, K., Molin, J., Bagribelsson, S. & Lindgren, B-M. | 2021 | ‘Acknowledge me as a capable person’: How people with mental ill health describe their experiences with general emergency care staff – A qualitative interview study | Emergency care | Semi-structured interviews | 11 |
| Eckerström, J., Flyckt, L., Carlborg, A., Jayaram-Lindström, N. & Perseius, K-I. | 2020 | Brief admission for patients with emotional instability and self-harm: A qualitative analysis of patients’ experiences during crisis | Mental health patients’ experiences with brief admission | Semi-structured interviews | 15 |
| Eldal, K., Veseth, M., Davidson, L., Skjølberg, Å., Gytri, D. & Moltu, C. | 2019 | Contradictory experiences of safety and shame in inpatient mental health practice – a qualitative study | Mental health hospitalization | Qualitative interviews | 14 |
| Hagen, J., Knizek, B.L. & Hjelmeland, H. | 2018 | Former suicidal inpatients’ experiences of treatment and care in psychiatric wards in Norway | Suicidal inpatients’ experiences of care in psychiatric wards | Qualitative interviews | 5 |
| Hipp, K. & Kangasniemi, M. | 2021 | Patient participation in pro re nata medication in forensic psychiatric care: Interview study with patients and nurses | Patient participation in forensic psychiatric inpatient care | Semi-structured interviews | 34 |
| Jones, A., Jess, K. & Schön, U-K. | 2021 | How do users with comorbidity perceive participation in social services? A qualitative interview study | User participation among patients with comorbidity of substance use and mental illness | Semi-structured interviews | 12 |
| Jørgensen, K.; Rasmussen, T.; Hansen, M.; Andreasson, K. & Karlsson, B. | 2020 | Recovery-oriented intersectoral care in mental health: As perceived by healthcare professionals and users. | Recovery-oriented intersectoral care | Focus group interviews | 9 |
| Klausen, R.K., Hansen Blix, B., Karlsson, M., Haugsgjerd, S. & Fagerjord Lorem, G. | 2017 | Shared decision making from the service users’ perspective: A narrative study from community mental health centers in northern Norway | Shared decision-making in mental health care | Qualitative interviews | 25 |
| Klevan, T., Jonassen, R., Topor, A. & Borg, M. | 2021 | Mutual learning: exploring collaboration, knowledge and roles in the development of recovery-oriented services. A hermeneutic-phenomenological study | Development and collaboration in recovery-oriented services | Semi-structured interviews | 8 |
| Koivisto, M., Melartin, T. & Lindeman, S. | 2021 | “If you don’t have a word for something, you may doubt whether it’s even real” – how individuals with borderline personality disorder experience change | Experiences of psychological change among patients with borderline personality disorder | Semi-structured interviews | 8 |
| Köpsén, S. & Sjöström, R. | 2020 | Patients’ experiences of a stress-management programme in primary care | Experiences of a primary care stress-management program | Semi-structured interviews | 9 |
| Lindberg, L.G., Johansen, K.S., Kristiansen, M. Skammeritz, S. & Carlsson, J. | 2021 | Negotiating engagement, worthiness of care and cultural identities through intersubjective recognition: Migrant patient perspectives on the Cultural Formulation Interview in Danish mental healthcare | Recognition among migrant patients in mental healthcare | Semi-structured interviews | 19 |
| Lindström, V., Sturesson, L. & Carlborg, A. | 2020 | Patients' experiences of the caring encounter with the psychiatric emergency response team in the emergency medical service—A qualitative interview study | Patients' experiences of the caring encounter with a psychiatric emergency response unit | Semi-structured interviews | 14 |
| Lockertsen, V., Nilsen, L., Wellhaven Holm, L.A., Rø, Ø., Burger L.M. & Røssberg, J.I. | 2020 | Experiences of patients with anorexia nervosa during the transition from child and adolescent mental health services to adult mental health services | Patients’ experiences of the transition between young/adolescent and adult mental health services | Semi-structured interviews | 10 |
| Lofthus, A-M., Westerlund, H., Bjørgen, D., Lindstrøm, J.C., Lauveng, A., Rose, D., Ruud, T. & Heiervang K. | 2018 | Recovery concept in a Norwegian setting to be examined by the assertive community treatment model and mixed methods. | Recovery in assertive community treatment | Mixed methods | 8 |
| Lofthus, A-M., Weimand, B.M., Ruud, T., Rose, D. & Heiervang, K.S. | 2018 | “This is not a life anyone would want”—A qualitative study of Norwegian ACT service users' experience with mental health treatment | Service users' experiences with the interprofessional Assertive Community Treatment (ACT) model | Focus groups and individual interviews | 8 |
| Møllerhøj J. & Os Stølan, L. | 2018 | ‘First and foremost a human being…’: user perspectives on mental health services from 50 mentally disordered offenders | Patient perceptions of their hopes and expectancies as well as their interactions with staff | Semi-structured interviews | 50 |
| Møllerhøj J., Os Stølan, L., Erdner, A., Hedberg, B., Stahl, K., Riise, J., Jedenius, E. & Rise, M.B. | 2020 | “I live, I don't work, but I live a very normal life”—A qualitative interview study of Scandinavian user experiences of schizophrenia, antipsychotic medication, and personal recovery processes | User experiences of schizophrenia, and their encounters with mental health services. | Semi-structured interviews | 24 |
| Nejati, S., Svenningsson, R.N., Björkelund, C. & Hange, D. | 2021 | How can a care manager at the primary care centre support foreign-born female patients suffering from common mental disorders? – An interview study | Experiences of patients with common mental disorders of health care encounters | Semi-structured interviews | 8 |
| Pedersen, M.K., Mohammadi, R. Mathiasen, K. & Elmose, M. | 2020 | Internet-based cognitive behavioral therapy for anxiety in an outpatient specialized care setting: A qualitative study of the patients’ experience of the therapy | Patients experiences of internet-based cognitive behavioral therapy while actively awaiting outpatient psychological treatment | Semi-structured interviews | 12 |
| Pelto-Piri, V., Wallsten, T., Hylén, U., Nikban, I. & Kjellin, L. | 2019 | Feeling safe or unsafe in psychiatric inpatient care, a hospital-based qualitative interview study with inpatients in Sweden | Psychiatric inpatient care | Qualitative interviews | 17 |
| Pettersen, H., Landheim, A., Skeie, I., Biong, S., Brodahl, M., Oute, J. & Davidson, L. | 2019 | How social relationships influence substance use disorder recovery: A collaborative narrative study. | Recovery and social relations among patients with substance use disorder | Semi-structured interviews | 18 |
| Røberg, L., Nilsen, L. & Røssberg, J.I. | 2018 | How do men with severe sexual and physical childhood traumatization experience trauma-stabilizing group treatment? A qualitative study | Gender-specific groups for trauma-stabilizing treatment. | Semi-structured interviews | 5 |
| Rønning, S.B. & Bjørkly, S. | 2017 | Residents’ experiences of relationships with nurses in community-based supported housing – a qualitative study based on Giorgi’s method of analysis and self-psychology | How residents in supported housing perceive their relationships with nurses. | Semi-structured interviews | 4 |
| Roos, E., Bjerkeset, O., Svavardottir, M.H. & Steinsbekk, A. | 2017 | Like a hotel, but boring: Users’ experience with short-time community-based residential aftercare | How patients with severe mental illness experience a stay in community residential aftercare | Qualitative interviews | 13 |
| Sather, E.W., Iversen, V.C., Svindseth, M.F., Crawford, P. & Vasset, F. | 2019 | Patients' perspectives on care pathways and informed shared decision making in the transition between psychiatric hospitalization and the community | Shared decision-making in psychiatric hospital centers and community mental health services | Focus group interviews | 10 |
| Sommerstad, H.S., Kildahl, A.N., Munkhaugen, E.K., Karlsen, K. & Bakken, T.L. | 2021 | Experiences of ward atmosphere in inpatients with intellectual disability and mental illness: Clinical implications for mental health nursing | Patients with intellectual disabilities in psychiatric services (comorbidity) | Semi-structured interviews | 10 |
| Stige, S.H., Binder, P-E., Stiegler, J.R., Schance, E., Andreassen Hummelslund, A. & Hjeltnes, A. | 2021 | Clients’ perspective on predetermined time limits for therapy in the context of the Norwegian welfare system | Psychotherapy within the context of the Norwegian welfare system | Semi-structured interviews | 18 |
| Sunnqvist, C., Berngarn, A., Ekezie, P.E., Lundgren, E. Nilsson, E. & Örmon, K. | 2022 | A pilot evaluation of a prehospital emergency psychiatric unit: The experiences of patients, psychiatric and mental health nurses, and significant others | Patients’ experiences of the treatment and care provided. | Qualitative interviews | 4 |
| Tarp, K., Hellum, R., Juhl Rasmussen, A. & Søgaard Nielsen, A. |  | Can creative writing, as an add-on to treatment for alcohol use disorder, support rehabilitation? | Patients with alcohol use disorder – self-perceived rehabilitating impact, improvement in quality of life, and executive functions. | Mixed methods  Interviews and observations | 6 |
| Tölli, S., Kontio, R.; Partanen, P. & Häggman-Laitila, A. | 2020 | Patient safety and staff competence in managing challenging behavior based on feedback from former psychiatric patients | Perceptions and experiences of former psychiatric patients of challenging behavior | Semi-structured interviews and video vignettes | 13 |
| Væggemose, U., Lou, S., Frumer, M., Limskov Stærk Christiansen, N., Aagaard, J. & Ørtenblad. L. | 2017 | Community families: Bridging the gap between mental health services and civil society – A qualitative study from users’ perspective | Users’ experiences, investments, and concerns of a befriending program | Semi-structured and focus group interviews | 20 |
| Waldemar, A.K., Esbensen, B.A., Korsbek, L., Petersen, L. & Arnfred. S. | 2018 | Recovery orientation in mental health inpatient settings: Inpatient experiences? | Inpatient services with a recovery-oriented approach | Semi-structured interviews | 14 |
| Wenaas, M., Wessel Andersson, H., Kiik, R. & Juberg A. | 2021 | User involvement in interprofessional team  meetings within services for substance use disorders | Patients’ experiences with interprofessional team meetings and to identify potential barriers to successful user involvement | Semi-structured interviews | 5 |
| Wergeland, N.C., Fause, Å., Weber, A.K., Beatrix, A., Fause, O. & Riley, H. | 2022 | Increased autonomy with capacity-based mental health legislation in Norway: A qualitative study of patient experiences of having come off a community treatment order | Patients experiences of user involvements | Qualitative interviews | 12 |
